# Unraveling Labrune Syndrome: A Case Report on the Neurological Phenotype in SNORD118-Negative Patients

**DOI:** 10.7759/cureus.62029

**Published:** 2024-06-09

**Authors:** Shalesh J Rohatgi, Siddharth R Nimal, Satish P Nirhale, Prajwal M Rao, Pravin U Naphade

**Affiliations:** 1 Neurology, Dr. D. Y. Patil Medical College, Hospital and Research Centre, Pune, IND

**Keywords:** cerebral microangiopathy, brain calcification, cysts, leukoencephalopathy, labrune syndrome

## Abstract

Labrune syndrome is a rare neurogenetic disorder with varied presentations. Here, we report the case of a 53-year-old male who presented with seizures, gait imbalance, and upper limb tremors for two years. Imaging studies revealed extensive leukodystrophy, multiple cerebral calcifications, and cystic lesions characteristic of Labrune syndrome. However, whole exome sequencing did not detect the *SNORD118* mutation, typically associated with Labrune syndrome. Although the *SNORD118 *mutation is commonly found in Labrune syndrome, a few cases of the syndrome without this mutation have also been reported. This suggests the possibility that other yet undiscovered mutations may cause the same phenotype.

## Introduction

Three cases of children with a wide range of clinical manifestations, including pyramidal, sensory, and cerebellar signs, were first reported by Labrune et al. [1]. The three main features of neuroimaging are hyperintense signals on T2-weighted imaging involving white matter, cerebral calcification, and cystic lesions. The primary pathology is cerebral microangiopathy, which coexists with micro-hemorrhage, cerebral calcification, and gliosis. In 2016, Jenkinson et al. identified the SNORD118 mutation that causes Labrune syndrome [2]. The *SNORD118 *gene may be essential for the maturation of ribosomal RNAs. In this paper, we report a case with clinical and radiological findings consistent with the diagnosis of Labrune syndrome but without the *SNORD118* mutation.

## Case presentation

**Figure 1:**
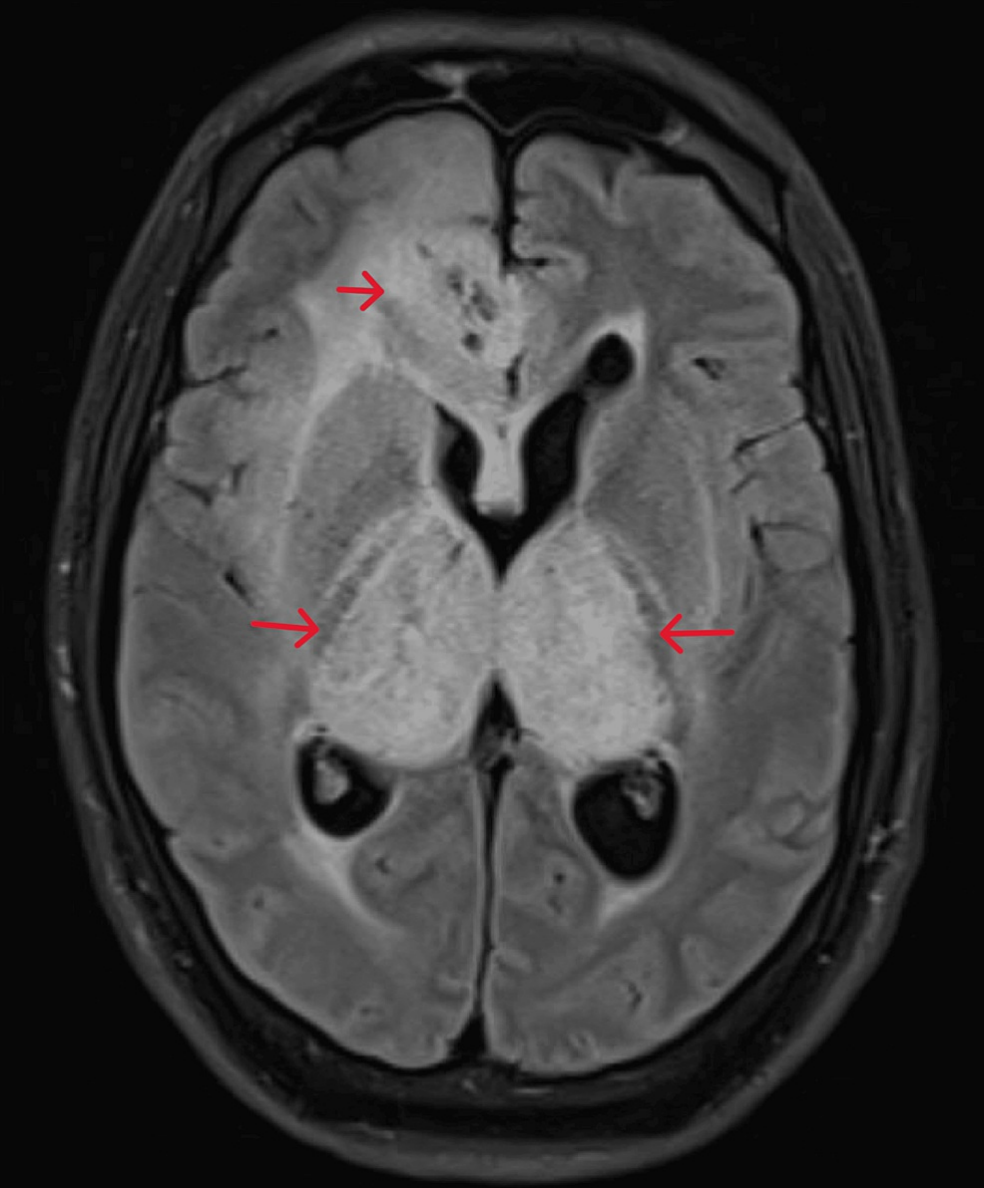
MRI brain fluid-attenuated inversion recovery (FLAIR) - Diffuse areas of altered signal intensities bilateral thalami, internal, and external capsules

**Figure 2:**
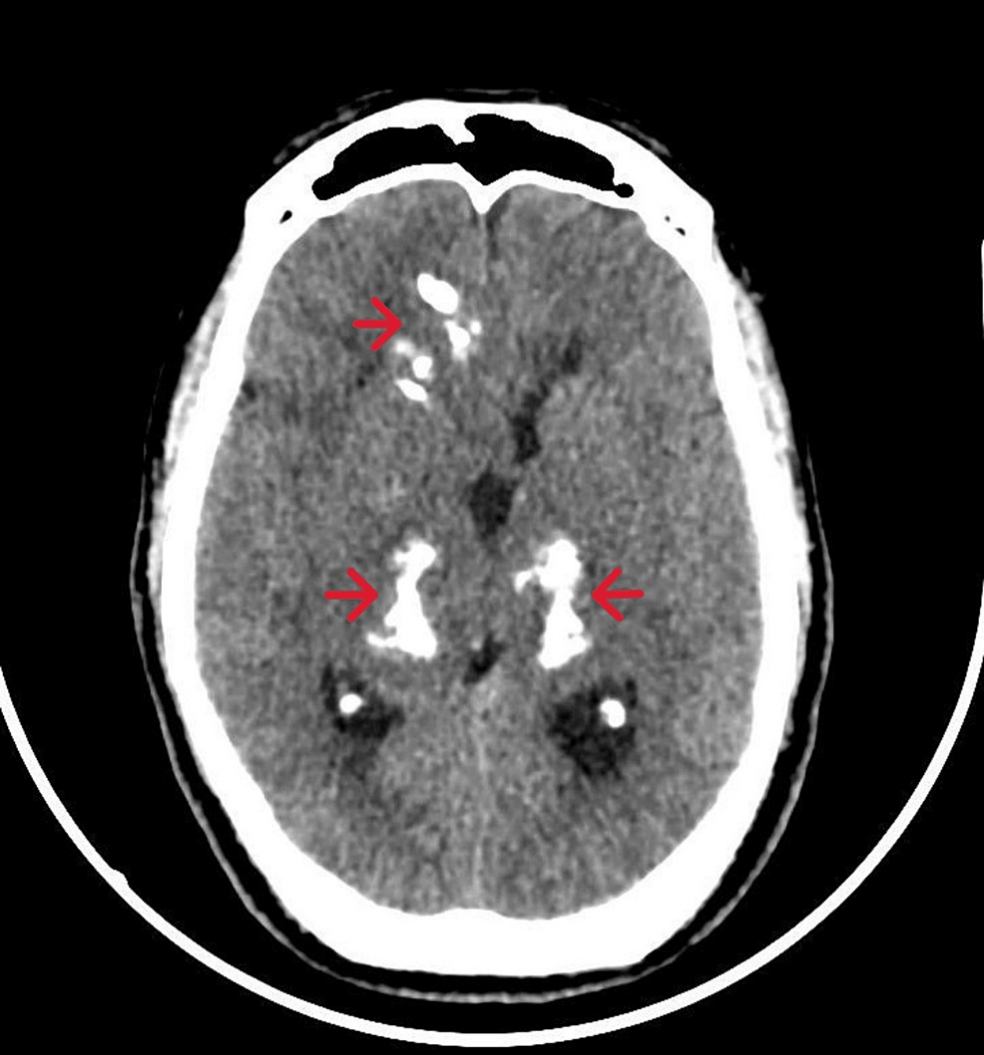
Brain CT - Diffuse areas of calcifications

A 53-year-old male with no known comorbidities presented with multiple episodes of focal seizures with impaired awareness, memory disturbances, imbalance while walking, and tremulousness of bilateral upper limbs subacute in onset and gradually progressive for two years. On neurological examination, he had mild to moderate cognitive deficits. Cranial nerve examination and fundus examination were normal. There was no gross sensory or motor deficit. Deep tendon jerks were normal, and the plantar reflex was flexor. Rhomberg's test was negative. Sensory examination was normal. He had bilateral cerebellar signs, including incoordination, finger-nose ataxia, dysdiadochokinesia, mild left-sided dysmetria, and gait ataxia. Routine blood investigations were normal. Brain CT revealed bilateral asymmetrical variable-sized coarse as well as punctate calcifications and edema involving subcortical white matter, deep nuclei, and brain stem (Figure 1). MRI brain revealed extensive areas of leukoencephalopathy, cerebral calcifications, and peripherally enhancing cystic lesions in the supra and infratentorial brain parenchyma (Figure 2-Figure 7). Some of these lesions, as in our case, were also present in the infratentorial brain parenchyma. Based on the clinical and radiological findings, a diagnosis of Labrune syndrome was considered. Whole-genome sequencing was requested, and no variants were identified in the *SNORD118 *gene. The patient was treated with antiepileptics and supportive care for seizures, along with physiotherapy and gait balancing exercises.

**Figure 3:**
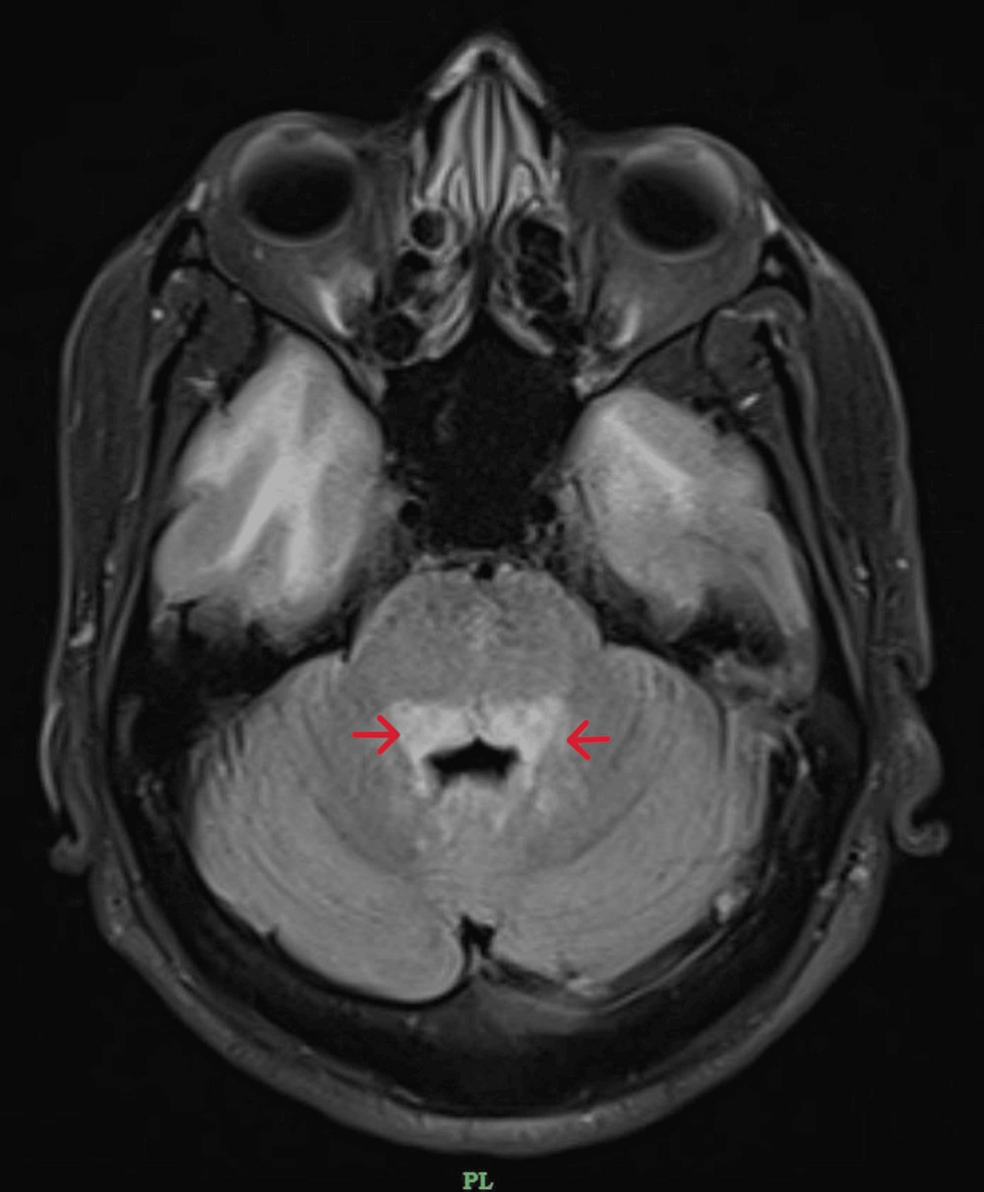
MRI brain fluid-attenuated inversion recovery (FLAIR) - Diffuse areas of altered signal intensities in dorsal midbrain pons superior and inferior cerebellar peduncles

**Figure 4:**
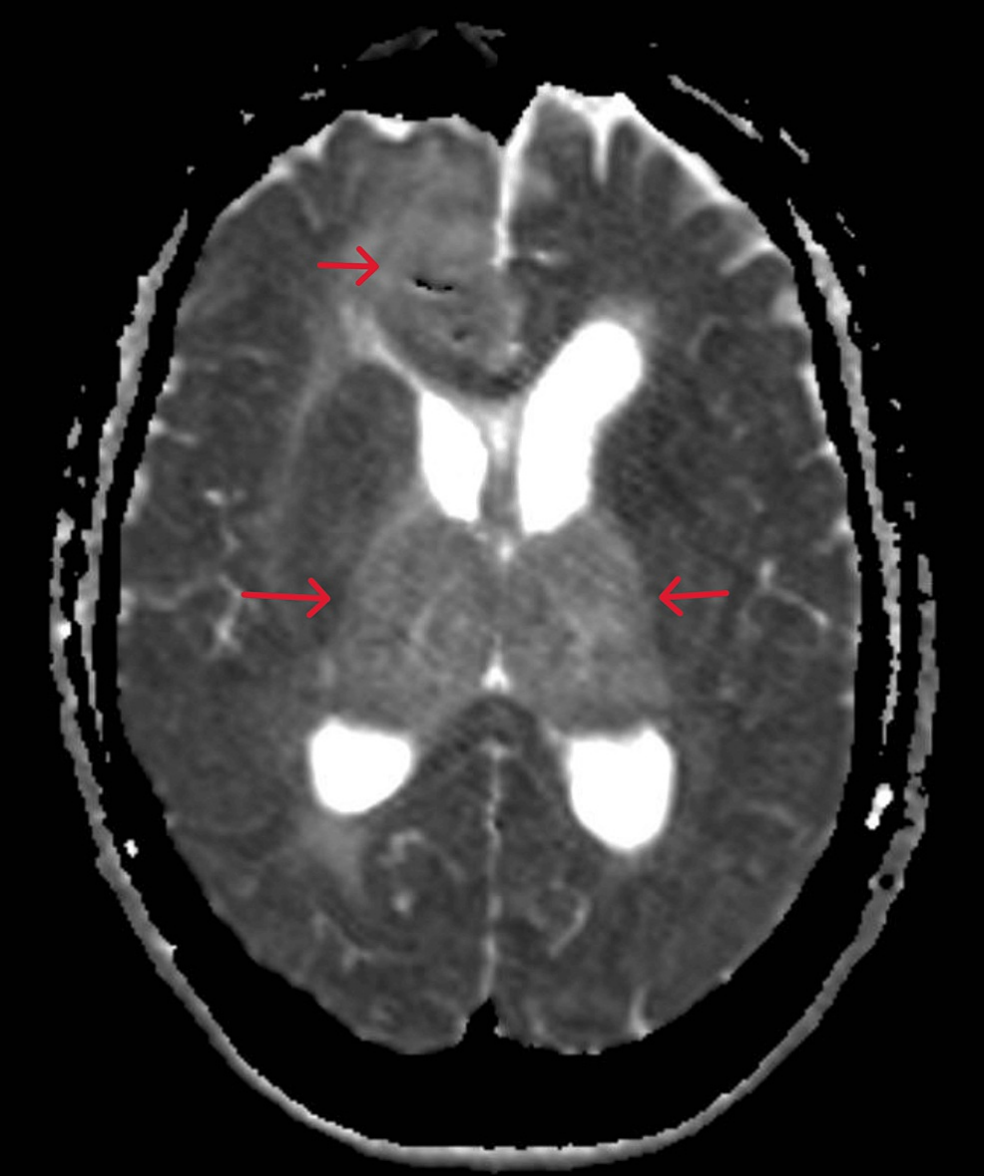
MRI brain T2 - Multiple ill-defined areas of altered signal intensity exhibiting variable T1 signal, low T2 signal noted within/adjacent to abovementioned lesions (diffuse areas of calcifications)

**Figure 5:**
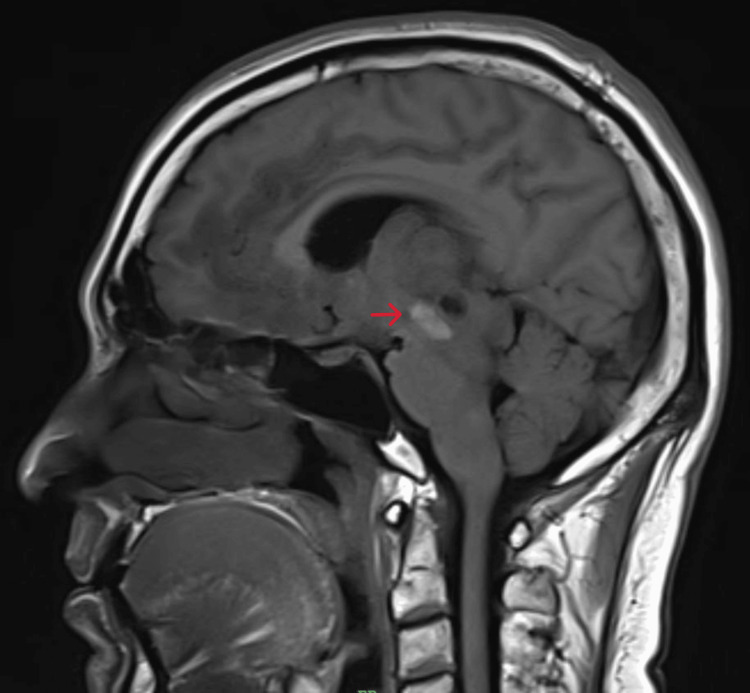
MRI brain T1 - Sagittal section: low T1 signal in infratentorial brain parenchyma

**Figure 6:**
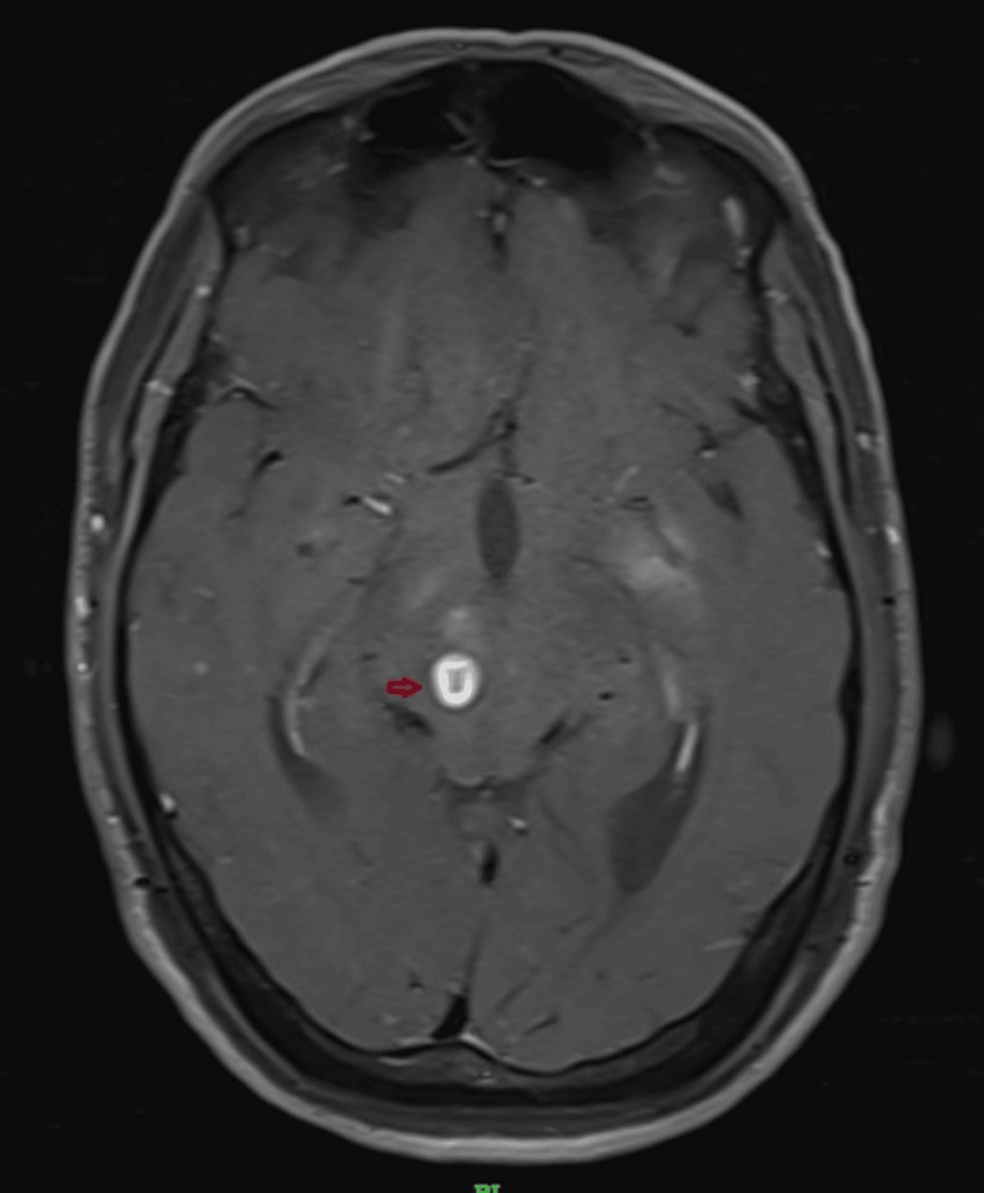
T1 - Post-contrast-axial - Peripherally enhancing cystic lesions

**Figure 7:**
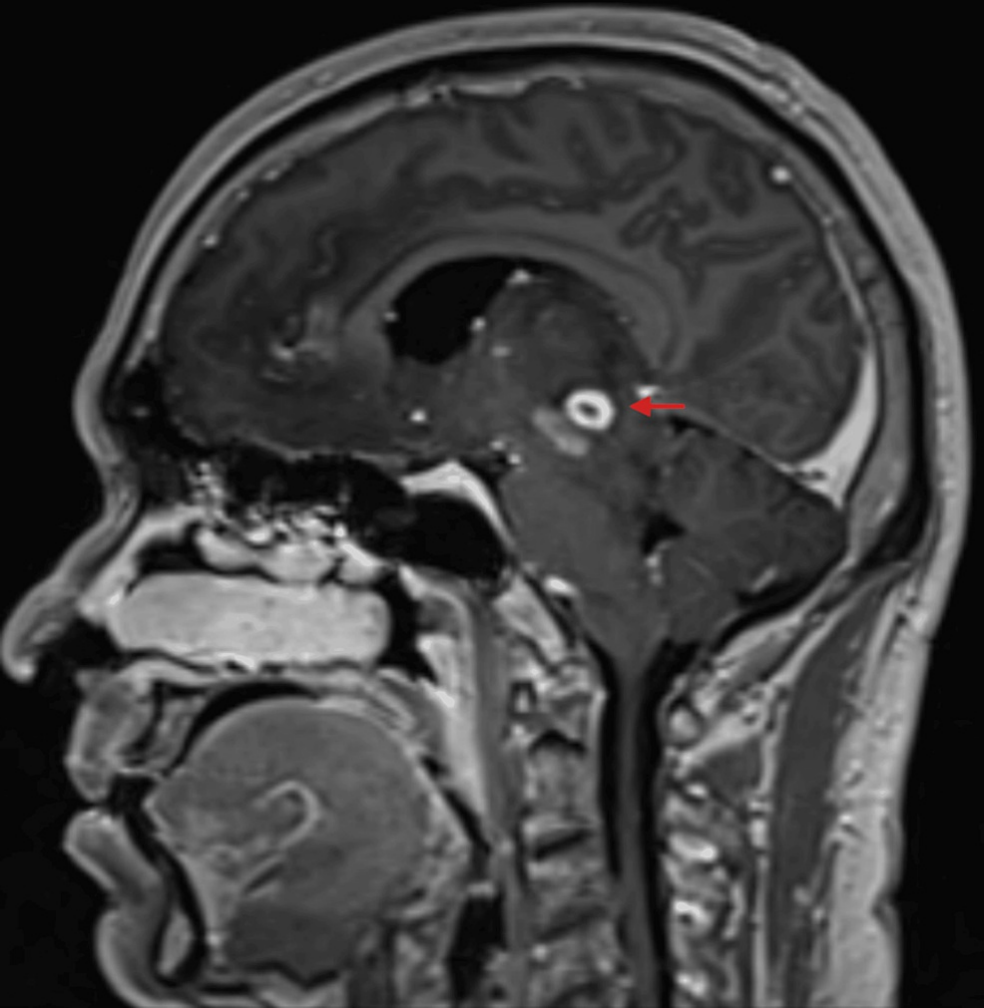
T1 - Post-contrast-sagittal - Peripherally enhancing cyst

## Discussion

Since Labrune and colleagues first described the triad of leukoencephalopathy with calcifications and cysts (LCC) in 1996, over 100 cases have been reported [1]. There is no defined protocol for diagnosis and treatment because of the uncommon occurrence [2]. T2 hyperintensity in the diffuse bilateral cerebral white matter is among the most observed neuroimaging features [3]. The corpus callosum is typically unaffected; however, damage may occur in the posterior fossa white matter. Brain calcifications are asymmetrically distributed and usually appear as small punctate foci or larger confluent regions within the cerebral white matter or deep grey nuclei. They are rarely located in the cerebellum [4]. They are typically found in the supratentorial region but can also affect the infratentorial region. In our case, the cysts were unevenly distributed within the brain parenchyma. The cause is believed to be microangiopathy, suggesting that LCC is more likely to be caused by disruption of the blood and brain barrier than by demyelination; hence, the term leukoencephalopathy may be misleading [5]. Although there are several infectious diseases, such as tuberculosis, toxoplasmosis, neurocysticercosis, cytomegalovirus, and tuberous sclerosis, that may be included in the radiologic differential, the diagnosis of LCC should be suggested by the triad of cerebral calcifications, parenchymal cysts, and diffuse white matter T2 hyperintensity without retinal or systemic involvement [6].

Coats plus syndrome (CPS) presents with the traditional triad of calcifications, cysts, and leukoencephalopathy; radiologically, it is indistinguishable from LCC. CPS is characterized by a *CTC1* gene mutation and retinal vascular disease. Coats disease is a congenital idiopathic retinal vasculopathy that was first discovered in 1908. It is characterized by retinal vascular telangiectasias and exudative retinopathy. Although this condition primarily affects young men, older patients have been reported to experience a less severe form known as adult-onset Coat's disease, which progresses at a slower rate. Exudative retinal detachment and the presence of subretinal and intraretinal exudates are typical clinical characteristics of this condition [7]. It is currently thought that the primary differences between LCC and CPS are that the latter does not exhibit a mutation in the *CTC1* gene, while the former's manifestation is limited to the central nervous system [7].

According to earlier research, a cyst wall biopsy could offer the highest diagnostic yield. A range of pathological features is present, including severe sclerosis with fibrinoid material deposited to the point of complete obliteration to mild mural hyalinization. Macrophages laden with perivascular hemosiderin were commonly noted alongside axonal loss and myelin pallor [7]. In the absence of definitive diagnostic criteria for LCC beyond its distinct radiological and clinical features, the decision was made to forgo a biopsy. This was due to the patient's reluctance to undergo the procedure, which was a limitation in this case.

As Labrune syndrome is so complex, it is essential to conduct thorough genetic investigations to discover any other genetic markers that may be connected to the disorder. This is especially true for patients who do not have the *SNORD118* mutation. The primary objective of current treatment modalities is to alleviate symptoms, which may involve surgically removing cysts, antiepileptic medications, or using antipsychotic drugs.

## Conclusions

We present an unusual case of cognitive decline, seizures, and tremulousness characterized by an imaging triad of LCC, also known as Labrune syndrome. The only way to differentiate it from other causes of LCC is through clinical evaluation, as it has no known systemic manifestations. Therefore, to fully understand the complex genetic foundations of Labrune syndrome and to develop specific treatment strategies, more genetic research is necessary.
